# HMGB1-Mediated Activation of the Inflammatory-Reparative Response Following Myocardial Infarction

**DOI:** 10.3390/cells11020216

**Published:** 2022-01-10

**Authors:** Eleonora Foglio, Laura Pellegrini, Matteo Antonio Russo, Federica Limana

**Affiliations:** 1 Technoscience, Parco Scientifico e Tecnologico Pontino, 04100 Latina, Italy; eleonora.foglio83@gmail.com; 2 Department of Experimental Medicine, Sapienza University of Rome, 00161 Rome, Italy; laura_pellegrini@hotmail.it; 3 IRCCS San Raffaele Roma and MEBIC Consortium, 00166 Rome, Italy; matteo.russo@sanraffaele.it; 4 San Raffaele University of Rome, 00166 Rome, Italy; 5 Laboratory of Cellular and Molecular Pathology, IRCCS San Raffaele Roma, 00166 Rome, Italy

**Keywords:** high mobility group box-1 protein, inflammatory and reparative response, myocardial infarction, molecular rehabilitation, cardiac repair

## Abstract

Different cell types belonging to the innate and adaptive immune system play mutually non-exclusive roles during the different phases of the inflammatory-reparative response that occurs following myocardial infarction. A timely and finely regulation of their action is fundamental for the process to properly proceed. The high-mobility group box 1 (HMGB1), a highly conserved nuclear protein that in the extracellular space can act as a damage-associated molecular pattern (DAMP) involved in a large variety of different processes, such as inflammation, migration, invasion, proliferation, differentiation, and tissue regeneration, has recently emerged as a possible regulator of the activity of different immune cell types in the distinct phases of the inflammatory reparative process. Moreover, by activating endogenous stem cells, inducing endothelial cells, and by modulating cardiac fibroblast activity, HMGB1 could represent a master regulator of the inflammatory and reparative responses following MI. In this review, we will provide an overview of cellular effectors involved in these processes and how HMGB1 intervenes in regulating each of them. Moreover, we will summarize HMGB1 roles in regulating other cell types that are involved in the different phases of the inflammatory-reparative response, discussing how its redox status could affect its activity.

## 1. Regulatory Role of Immune System in Post-MI Inflammation and Healing

The repair process following acute myocardial infarction (AMI) is dependent on an optimally orchestrated inflammatory response, which involves the immune system at multiple levels, and is mediated by cytokines and inflammatory cells that infiltrate the infarcted myocardium. The main goal of the inflammatory response is firstly to remove dead cells and matrix debris by phagocytosis and, later, to provide key molecular signals for the activation of reparative cells, therefore promoting tissue repair and scar formation [1]. The inflammatory response is a key determinant of the final infarct size and of the recovery of heart function and, for this reason, recently it has become an important target for cardioprotection [2,3]. Cardiac repair after AMI consists of three overlapping phases: an early inflammatory phase (first 72 h after AMI); a late reparative and proliferative phase (4–7 days post AMI); and a maturation phase (from 7–10 days post AMI up to several months) [4,5]. For each of these phases, the intervention of the immune system is crucial.

The predominant mechanism of cardiomyocyte death in the infarcted heart is represented by necrosis. The initial acute inflammatory response to AMI is triggered by the innate immune system. Cell necrosis and destruction of the extracellular matrix (ECM) generate damage-associated molecular patterns (DAMPS) that serve as danger signals [6,7] and lead to activation of immune cells [5]. DAMPS bind to pattern-recognition receptors (PRRs) present on neutrophils and activate the complement cascade that propagates the inflammatory signaling by inducing the production of cytokines, chemokines, and adhesion molecules [8]. The innate immune response activates and triggers the accumulation of immune cells into the ischemic myocardium with the purpose to clear necrotic cell debris from the infarcted zone: the interaction between cell adhesion molecules on endothelial cells and their receptors on leukocytes leads to the recruitment and extravasation of neutrophils and mononuclear cells into the infarcted myocardial tissue [9]. Specifically, there is an early infiltration of neutrophils into the infarcted zone (from 6 to 24 h post-MI), followed by the accumulation of pro-inflammatory monocytes and macrophages (over the next 48–72 h) [3]. Phagocytosis, exerted by both neutrophils and macrophages, is critical for the removal of debris from the infarcted area and for an adequate scar formation.

The inflammatory phase is followed by a reparative and proliferative phase that allows wound healing and proper scar formation to prevent myocardial rupture and partially limit functional deterioration. This phase is mainly driven by anti-inflammatory monocytes/macrophages and is mediated by suppression, resolution, and containment of the initial pro-inflammatory response in addition to fibroblast proliferation and deposition of granulation tissue [10]. Inhibition and resolution of post-infarction inflammation are active processes that require the activation of multiple inhibitory pathways coordinated by the action of several different cell types (e.g., neutrophils, mononuclear cells, endothelial cells, and pericytes) and by cardiac fibroblast activation, leading to alterations in ECM composition [11]. An important process that begins during this phase is represented by angiogenesis, characterized by the formation of new functional blood vessels from pre-existing capillaries that provide oxygen and nutrients to the highly dynamic and metabolically active cells of the healing wound. Finally, there is a maturation phase associated with the remodeling of the ECM that lasts several months. This phase strictly depends on the evolution of the previous phases and is critical for the restoration of heart function. An unsuccessful maturation phase may lead to an increase in myocardial stiffness, diastolic dysfunction, and development of heart failure (HF) [5].

After MI, infarct expansion occurs and triggers hypertrophy and dilatation of the left ventricle (LV), thereby inducing a progressive increase in LV volume and a reduced LV ejection fraction (EF), eventually leading to chronic HF. This process, characterized by changes in LV size and function, is referred as LV remodeling. An excessive inflammatory response after MI may interfere with the healing process, thereby exacerbating post-MI LV remodeling and resulting in an extension of the inflammatory infiltrate into the non-infarcted myocardium that induces the activation of pro-apoptotic pathways with further loss of cardiomyocytes, augmented matrix degradation, and impaired collagen deposition. All of these processes lead to the formation of a scar with reduced tensile strength more susceptible to rupture along with enhanced fibrosis and worsened diastolic function [11,12]. Nevertheless, numerous evidence demonstrated that post-MI treatment with anti-inflammatory drugs (e.g., steroids) increased the incidence of cardiac rupture [13], suggesting that inflammation is a double-edged sword [14] and is very often considered “harmful” in an oversimplified manner because, without a proper initial inflammatory phase, severe left ventricular dysfunction is the logical consequence of myocardial infarction. Thus, elucidation of the mechanisms that regulate the inflammatory phase will contribute to the development of new therapeutic strategies designed to control excessive inflammatory response, while promoting the physiological healing process following MI.

## 2. HMGB1 as a DAMP

As previously mentioned, the acute inflammatory response leads to the generation of DAMPS that, in turn, trigger the activation of the inflammatory response. The archetype DAMP is HMGB1 [15], a highly abundant and evolutionary conserved protein expressed in almost all eukaryotic cells [16] that has been demonstrated to have several important biological activities inside and outside the cell.

### 2.1. Structure and Localization

**Figure 1:**
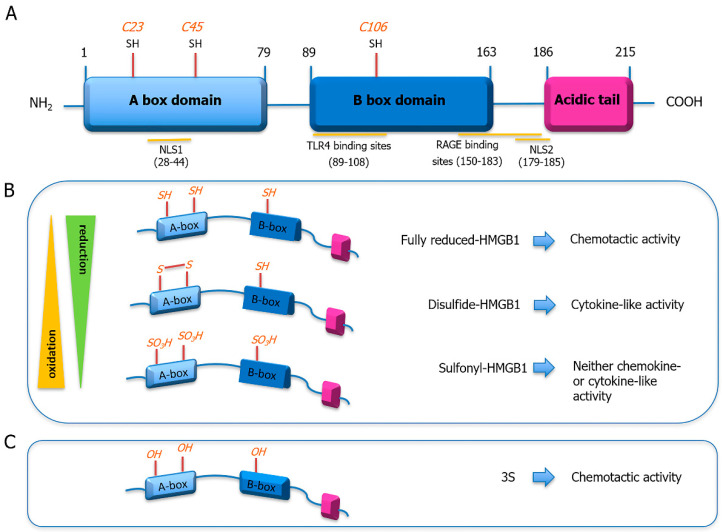
HMGB1: structural characteristic and redox modifications. (**A**) HMGB1 is a 215-amino acid protein of 30 kDa that comprises three domains: two positively charged domains (A Box and B Box) and a negatively charged carboxyl terminus (acidic tail). Each domain has peculiar features. (**B**) HMGB1 exists in three redox forms. The fully reduced HMGB1 is characterized by all the three cysteines in the thiol state and exerts chemotactic activity. The partial oxidation of HMGB1 leads to the formation of an intramolecular disulfide bond between the C23 and C45 and defines the disulfide-HMGB1 that acts as a pro-inflammatory cytokine. The further oxidation of all cysteines to sulfonates characterizes the sulfonyl HMGB1 that has neither chemokine- nor cytokine-like activity. (**C**) Recombinant 3S is characterized by the substitution of cysteines with serine residues.

HMGB1 is composed of 215 amino acid residues organized in three structural domains: two positively charged homologous DNA-binding domains, named A Box and B Box, and a highly negatively charged C-terminal tail [17] (Figure 1A). The A and B boxes are responsible for the binding of DNA [18] while the C terminus mainly regulated the DNA binding/bending and is essential to assure HMGB1 proper function through the maintenance of its stability [19,20].

HMGB1 continually shuttles between the cytoplasm and the nucleus with its equilibrium shifted towards the latter in physiological condition [21]. Two lysine-rich nuclear localization signals (NLS), located in the A Box domain (aa 28–44) and between the B Box and the C tail (aa 179–185), respectively, and two non-classical nuclear exportation signals (NES), present in each of the DNA binding domains, are involved in this activity [21]. In particular, the post-translational modifications occurring in these regions, in activated or stressed cells, are responsible for the translocation of HMGB1 from the nucleus to the cytoplasm and for the rate at which this process proceeds [22]. The acetylation of the lysine residues in the NLSs by P300/CBP-associated factor (PCAF), CREB binding protein (CBP), or histone acetyltransferase p300 (p300) [23] promotes the relocation of HMGB1 in the cytoplasm by reducing its binding to the nuclear importin protein karyopherin-1 [21], and prevents its return into the nucleus. Translocation of HMGB1 is also regulated by oxidation and phosphorylation. HMGB1 has three conserved redox-sensitive cysteines, two in the A Box, C23 and C45, and C106 in the B Box. In the process of nucleocytoplasmic shuttling of HMGB1, C106 appears to be critical, indeed its mutation impairs HMGB1 nuclear distribution [24]. The phosphorylation of serine residues within the NLSs [25], catalyzed by the calcium/calmodulin-dependent protein kinase type IV (CaMKIV) or the classical protein kinase C (cPKC) [26,27], is also essential to the translocation of HMGB1 in the cytoplasm. Once there, HMGB1 can be moved to the cellular membrane [28] or actively secreted, following stress stimuli, in the extracellular space via a non-classical vesicle compartment-mediated secretory pathway [29]. When actively secreted, HMGB1 is hyper acetylated as a consequence of pro-inflammatory stimuli [30]. Nonetheless, HMGB1 can be found in the extracellular space also due to the plasma membrane rupture in damaged or necrotic cells. Finally, HMGB1 can be found on the cellular surface or in microparticles or exosomes and through the use of electron microscopy and immunofluorescence techniques it has been observed in mitochondria and peroxisome in neuron cells after ischemic insult [31].

### 2.2. Functions

Based on its localization, post-translational modification and context, HMGB1 exerts different functions. In the nucleus, as a result of its binding/bending activity and of its interaction with the nucleosomes, HMGB1 plays an important role in the regulation of gene transcription [32,33] and DNA repair [34]. In addition, it is involved in the modulation of DNA replication [35], V(D)J recombination [36], telomerase activity and function [35,36,37] and, by inducing the expression of the heat shock protein 27 (HSP27), HMGB1 participates in the autophagic process [38].

In the cytoplasm the main function of HMGB1 is the regulation of autophagy that is achieved by the binding of the HMGB1 intramolecular disulfide bridge between C23 and C45 to Beclin1 that leads to the disruption of the interaction between Beclin1 and Bcl2 [39]. In tumor cells, moreover, it has been reported that HMGB1 can bind mitochondrial DNA (mtDNA) released following hypoxic stimuli and activates toll like receptor 9 pathway to promote cellular proliferation [40].

Moreover, HMGB1 can mediate platelet activation, neurite outgrowth, innate immunity and erythroid maturation and proliferation when it is localized on the cell membrane (reviewed in [23]).

However, most of the HMGB1 pleiotropic functions are exerted in the extracellular space and are mainly defined by its form (monomer, dimer, multimer), concentration, interactions with different molecules or receptors, and by the oxidation status of its cysteine residues (i.e., reduced state (fr-HMGB1), disulfide form (ds-HMGB1), oxidized state (ox-HMGB1)) (Figure 1B) [41]. HMGB1 can be involved in inflammation, migration, invasion, proliferation, differentiation, and tissue regeneration but it can also be responsible for the deleterious effects observed in contexts like diabetes [42], ischemia-reperfusion injury [43], or sepsis.

### 2.3. Receptors and Signaling

The C-X-C chemokine receptor type 4 (CXCR4), a G protein-coupled receptor (GPCR), is also involved in mediating the chemotactic activity of fr-HMGB1. However, HMGB1 does not bind directly to CXCR4, but requires the formation of a heterocomplex with CXCL12, consisting of one molecule of HMGB1 and two molecules of CXCL12, each interacting with one HMG-box domain [44]. The heterocomplex protects CXCL12 from degradation and its binding to CXCR4 determines the migration of different cell types as macrophages, dendritic cells, mouse embryonic fibroblasts, human cardiac fibroblasts and myoblasts by the activation of a signaling cascade that involves ERK phosphorylation and Ca^2+^ release from cellular stores [44].

The Advanced Glycation End products Receptor (RAGE) has been the first HMGB1 receptor to be identified [45]. It is a transmembrane receptor expressed on a wide variety of cells, able to recognize different proteins, such as advanced glycation end products (AGEs) and ECM components, among others (reviewed in [46]). Once fr-HMGB1 binds to RAGE, it activates multiple signaling molecules, including ERK ½ [47], p38 [48], the Rho family small GTPase CDC42/Rac [49], Src [50], and NF-κB, promoting cell migration, proliferation, differentiation, and adhesion and stimulating the expression of cell surface receptors [23]. The activation of these pathways has been frequently observed in different pathologies in which HMGB1 plays an important role, such as cancer, sepsis, neurological and cardiac diseases, suggesting how this axis could represent an important therapeutic target. RAGE can interact also with ds-HMGB1, leading to an increased transcription of the chemotactic gene stromal derived factor 1 (SDF-1) also known as CXCL12 [41]. Moreover, this binding is necessary for platelet-dependent neutrophil activation and for the formation of neutrophil extracellular traps (NETs) in the thrombo-inflammatory lesions [51].

The Toll-like receptor 2/4 (TLR2/4), although able to bind fr-HMGB1 [52], is triggered only by ds-HMGB1 through the formation of a complex with CD14 and a TLR4 adaptor, the myeloid differentiation factor 2 (MD-2). It is not known, instead, which HMGB1 redox form activates TLR2 and whether the binding is direct or mediated by other proteins. However, the interaction of HMGB1 with TLRs induces the nuclear translocation of NF-κB with the following expression of pro-inflammatory cytokines and inflammatory mediators through the TIR-domain-containing adapter interferon-γ (TRIF), and the myeloid differentiation primary-response protein 88 (MyD88) dependent pathways [53] in neutrophils and macrophages. In the heart, the TLR4/HMGB1 pathway has been demonstrated to mediate inflammatory and injurious responses associated with heart diseases, in particular regulating the apoptotic death of cardiomyocytes [54].

## 3. Role of HMGB1 in the Inflammatory and in the Reparative Phases following MI

**Figure 2:**
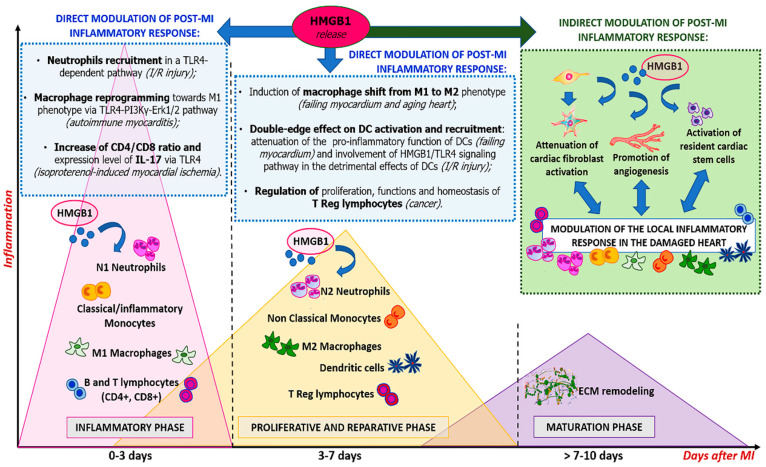
Role of extracellular HMGB1 in modulation of post-MI inflammatory response. HMGB1 has recently emerged as a possible regulator of the inflammatory reparative response (IRR) after AMI. Specifically, HMGB1 directly modulates the activity of different immune cell types involved in the different phases of IRR after MI, both when a pro-inflammatory environment is predominant (in the early inflammatory phase) and also when it is progressively replaced by the anti-inflammatory milieu that favors heart regeneration and repair (in proliferative/reparative and maturation phases). Moreover, HMGB1 influences post-MI inflammatory response also by recruiting endogenous stem cells, by activating endothelial cells to induce angiogenesis and by modulating cardiac fibroblast activity, all processes that, in turn, directly or indirectly involve immune cells.

As previously described, cells of the immune system are present in the infarcted myocardium at all stages of the heart repair process. Many cell populations have been shown to manifest both inflammatory and anti-inflammatory properties depending on the signals received from the microenvironment and, very often, their timely switch from an inflammatory to an anti-inflammatory activity is crucial during post-MI myocardial healing. Here, we will focus on the main cellular effectors involved in the inflammatory repair response following cardiac damage and we will describe the role of HMGB1 in their regulation (Figure 2).

### 3.1. Neutrophils

Neutrophils are polymorphonuclear myeloid cells specialized in removing invading pathogens by phagocytosis with very high efficiency and killing them through the production of reactive oxygen species (ROS) or the release of proteases and other biologically active substances present in their granules [55,56].

In the first few hours following MI, endothelial cells (ECs) trigger vessel loss integrity and increased permeability to allow neutrophil infiltration: due to their extreme motility, neutrophils are the first cells of the immune system to infiltrate the site of infarction, where they coordinate the initial phase of the inflammatory response [57]. In the mouse model of acute MI, the number of neutrophils peaks at days 1–3 post-MI and, by one month after MI, there is a low-grade inflammation and only few neutrophils are present in the infarcted region [58].

Once migrated into the infarcted area, recruited neutrophils clear the site of myocardial injury, mainly through phagocytosis of cellular debris, rapid degranulation, release of matrix-degrading enzymes, production of ROS, and activation of a recently described process, called NETosis, characterized by the release of chromatin fibers into the extracellular space and the formation of neutrophilic extracellular traps (NETs) [59]. Further, neutrophils propagate the acute inflammatory response to neighboring areas by expressing mediators capable of amplifying cell recruitment and triggering monocyte infiltration into the ischemic tissue [60]. Unfortunately, this process is also associated with further tissue destruction [61]. It is noteworthy that an elevated number of neutrophils is associated with bad prognosis in MI patients [62]. Further, neutrophil depletion in animals undergoing temporary coronary artery occlusion has been reported to significantly decrease the size of the infarct, confirming that myocardial injury may be induced by neutrophil-dependent mechanisms [63,64]. In the context of myocardial I/R, HMGB1 plays a central role in recruiting neutrophils in a TLR4-dependent pathway worsening myocardial injury [43]. Accordingly, in a TLR4-mutant mouse model, the induction of 30 min ischemia followed by 6 h reperfusion determined a significant inhibition in the expression of TNFα, IL8, and HMGB1 and the recruitment only of few neutrophils with tissue structure preservation [65].

Nevertheless, the recruitment of neutrophils has been shown to be required for a successful switch from the inflammatory response to the reparative phase of cardiac healing following MI, highlighting also a potential protective role for neutrophils [66]. First of all, the abundant neutrophils infiltrating the infarcted myocardium are short-lived inflammatory cells programmed to undergo apoptosis after degranulation in order to prevent prolonged inflammation: 3–7 days after the acute event, in fact, the neutrophil infiltrate resolves in both mouse and canine infarcts [67] as most granulocytes undergo apoptosis. Moreover, dying neutrophils release mediators, such as annexin A1 and lactoferrin, that further inhibit neutrophil recruitment [68] and also act as chemoattractant for anti-inflammatory macrophages with the capacity to phagocytize them. Secondly, it has been demonstrated that the uptake of apoptotic neutrophils can polarize macrophages towards a reparative phenotype [69], leading to the production of anti-inflammatory cytokines such as transforming growth factor beta (TGF-β) and IL-10 [70].

Recently, it has been shown that neutrophils directly contribute to the reparative phase [71]. Daseke and colleagues have shown that neutrophils can polarize after MI, exhibiting a pro-inflammatory N1 phenotype during the inflammatory phase (at days 1–3 post-MI) and an anti-inflammatory N2 phenotype (CD206^+^) during the reparative phase (at days 3–7 post MI) [58,72,73]. Furthermore, 5 and 7 days following MI, neutrophils produce ECM proteins (including fibronectin, vimentin, and fibrinogen) necessary for scar formation.

### 3.2. Monocytes/Macrophages

Monocytes, produced in the bone marrow and spleen, enter the bloodstream and are recruited to the infarcted zone in the first few hours following MI. Infiltration of monocytes into the infarcted myocardium is followed by their maturation and differentiation into macrophages.

In mice, most of the monocytes that migrate to the site of infarction (peaking at day 3 after MI) is represented by pro-inflammatory Ly6C^high^, while a minor part is represented by Ly6C^low^. Ly6C^high^ monocytes, which are referred as classical/inflammatory monocytes due to their ability to extravasate into tissues, highly express CCR2, TNF-α, and IL-1b; further, they produce and secrete different growth factors, such as FGF-2 and VEGF and secrete matrix metalloproteinases (MMPs), such as MMP-9 [74], all of which promote angiogenesis [75]. Monocyte subpopulations with distinct modulatory effects on inflammatory responses have also been described in humans, namely classical CD14^++^CD16^−^ monocytes, intermediate CD14^++^CD16^+^ monocytes, and non-classical CD14^+^ CD16^++^ monocytes. Human classical CD16^−^ monocytes express high levels of CCR2 and have pro-inflammatory properties resembling murine Ly6C^high^ cells [76]. Circulating pro-inflammatory CD14^++^/CD16^−^ cells showed an early peak in patients with ST elevation myocardial infarction and were negatively associated with recovery of function [11,77].

In the pro-inflammatory environment of the healing infarct, upregulation of Macrophage Colony Stimulating Factor (M-CSF) induces monocytes to differentiate into macrophages [78]. The Ly6C^high^ monocytes differentiate into classically activated pro-inflammatory macrophages (M1 or CCR2^+^) that express IL-1b and TNF-α [79]. These macrophages scavenge debris and secrete inflammatory cytokines and matrix-degrading proteases [75]. During resolution of post-infarction inflammation, instead, a smaller subset of Ly6C^low^ monocytes and alternatively activated M2 (or CCR2^−^) macrophages become the predominant subtypes [80] and promote the healing response to MI by secreting mediators that suppress inflammation and increasing the phagocytic activity for the removal of inflammatory leukocytes. Moreover, they contribute to collagen deposition to form scar tissue that replaces lost cardiomyocytes in the infarcted zone and to angiogenesis [75,79].

Macrophages represent numerically the predominant cells infiltrating the infarcted myocardium, with M1 macrophages (those related to pro-inflammatory processes) dominating at 1–3 days post-MI, whereas M2 macrophages (those involved in resolution and repair) becoming the predominant macrophage subset 5 days following MI [81].

Generally, different pools of cytokines seem to be associated with the distinct phases of inflammation and fibrotic remodeling after ischemia. TNF-α, IL-1β, and IL-6 are the most secreted cytokines during the inflammatory phase by M1 macrophages, whereas IL-10, TGF-β, PDGF, and tissue inhibitors of metalloproteinases (TIMPs) belong to the secretome of alternatively activated macrophages (M2) and are fundamental for the generation of myofibroblasts [82] and for the transition from the inflammatory to the proliferative phase [83]. Once in the infarcted area, macrophages play a role in post-MI fibrosis, matrix remodeling, and angiogenesis.

Several studies demonstrated an involvement of HMGB1 in modulating macrophage polarization toward both phenotypes in different cardiac diseases. For instance, in experimental autoimmune myocarditis, HMGB1 facilitated macrophage reprogramming towards a pro-inflammatory M1 phenotype via TLR4-PI3Kγ-Erk1/2 pathway, as demonstrated by a reduction in infiltrating M1 macrophages following HMGB1 stimulus and TLR4 blockade, PI3Kγ inhibition, or Erk1/2 inhibition [84].

In contrast, another study demonstrated that extracellular HMGB1 is also able to recruit monocytes at the site of tissue damage and coordinate the switch of macrophages to a tissue-healing phenotype [85]. Specifically, using a rodent model of heart failure, the group of Dr. Suzuki showed that extracellular HMGB1 contributed to the protective effects exerted by bone marrow mononuclear cells (BMCs) following transplantation in the failing myocardium by modulating macrophage’s polarization towards the anti-inflammatory M2 phenotype [85]. HMGB1-inhibition, by an anti-HMGB1 antibody, abolished the enhancement of CD63^+^ M2 macrophages and exacerbated the increase in CD86^+^ M1 macrophages. Further, IL10, known to be secreted by activated M2 macrophages, resulted highly expressed by RT-PCR following BMC transplantation, but totally repressed by inhibiting HMGB1. These results suggested a role of HMGB1 in macrophage shift from M1 to M2 phenotype.

The modulation of macrophage polarization by HMGB1 has been involved also in the aging heart [86]. Senescence-accelerated prone mice (SAMP8 mice) represent a murine model of spontaneous senescence [87]. By immune-histochemical staining, these mice showed a decrease in cardiac M2 macrophages compared to control mice, as demonstrated by the downregulation of two M2 macrophage specific markers, i.e., CD36 and IL-10. On the contrary, the protein expression of CD68, the specific M1 macrophage marker, along with HMGB1, TLR2, and TLR4 (HMGB1 cascade proteins) were significantly increased in SAMP8 mice compared to controls. These results prompted the authors to hypothesize that HMGB1-TLR2/TLR4 cascade and the induction to M1 macrophage polarization might eventually lead to cardiac dysfunction in aged hearts.

Interestingly, the immunosuppressive protein C1q has been recently demonstrated to inhibit the proinflammatory effects of HMGB1 on monocytes by forming a tetramolecular complex comprising HMGB1, RAGE, and LAIR-1 (high-affinity receptor for C1q) and directing monocytes to an anti-inflammatory phenotype unable to differentiate to dendritic cells (DCs) [88].

### 3.3. Dendritic Cells (DCs)

Ly6C^high^ monocytes also give rise to dendritic cells (DCs), depending on the local tissue environment. DCs show a strong antigen-presenting capacity and play a pivotal role in innate immunity by controlling the excessive inflammatory response in sterile inflammation via the expression of the anti-inflammatory cytokine IL-10. Further, they are also fundamental in acquired immunity, by producing chemokines such as IFN-γ and thus regulating immune cell trafficking and promoting T cell activation [89].

The role of DCs in cardiovascular diseases is controversial [14]. After MI, DCs are induced to differentiate from the same precursor cells as monocytes/macrophages and infiltrate the infarction site, which peaks at 7 days post-MI [81]. These cells control the excessive inflammatory response that develops after MI, by expressing the anti-inflammatory cytokine IL-10 and by regulating the homeostasis of monocytes and macrophages, favoring the transition from inflammation to repair [90,91]. Accordingly, DC-ablated infarcted mouse hearts were characterized by a marked infiltration of inflammatory monocytes and M1 macrophages and by an impaired recruitment of anti-inflammatory monocytes and M2 macrophages compared to control mice. Further, the expression of inflammatory cytokines as well as MMP-9 activity increased, while IL-10 expression decreased [91].

Furthermore, it has recently been shown that administration of heart-specific tolerogenic DCs (primed with lysate from infarcts) resulted in attenuated ventricular remodeling, preserved left ventricular systolic function and improved survival, by inducing regulatory T cells (Tregs), which elicited an inflammatory-to-reparative macrophage shift [92]. Thus, targeting DCs could be an alternative therapeutic strategy to stimulate the beneficial action of Tregs and improve cardiac remodeling in post-AMI patients.

However, in contrast, in a rat model of MI, an increased number of mature DCs in the infarcted heart has been found to be associated with deterioration of LV remodeling [90].

The effect of HMGB1 on DC activation and recruitment may also be double-edged. An early study showed that exogenous HMGB1 attenuated the proinflammatory function of DCs by inhibiting the secretion of pro-inflammatory cytokines [93]. Further, in a rodent model of post-MI chronically failing heart, the improvement in cardiac function following intramyocardial injection of HMGB1 was associated with attenuated accumulation of DCs [94]. Nevertheless, several reports have demonstrated the pro-inflammatory properties of extracellular HMGB1 on DCs [95,96]: HMGB1 finely tunes the maturation, Th1 polarization, and immune functions of this subset of cells [95] and is required for the migration of maturing DCs [96] in a RAGE dependent manner. Interestingly, a recent study showed HMGB1-mediated detrimental effects of DCs in the setting of myocardial I/R injury [97]. Antagonizing HMGB1 by a specific neutralizing antibody or blocking TLR4 soon after myocardial I/R determined reduction in the adhesion and aggregation of DCs, inhibition of costimulatory molecule expression and decreased inflammatory mediator release aggravating cardiac function. These results demonstrated the involvement of HMGB1/TLR4 signaling pathway in the detrimental effects of DCs in the process of I/R.

Another group demonstrated that, following I/R injury, necrotic cardiomyocytes released both HMGB1 and cell-free DNA (cfDNA) that entered into the circulation, activated the spleen to exacerbate the inflammatory response and, eventually, worsened tissue damage during reperfusion by a common RAGE-Toll-like receptor 9-dependent mechanism [98]. Interestingly, the same group, in a most recent study, hypothesized that cfDNA and HMGB1 stimulated plasmacytoid dendritic cells (pDCs) to secrete type I interferon (IFN-I) that amplified tissue injury during reperfusion [99].

### 3.4. Components of the Adaptive Immune System

A role for T and B lymphocytes in the inflammatory response to MI has recently emerged too, although a lower lymphocyte count has been observed when compared with neutrophils and monocytes [100].

An increased number of peripheral B lymphocytes has been reported in patients with MI [89,101]. Recent studies showed that B lymphocytes are recruited to the injured myocardium after MI, where they promote CCL7-mediated pro-inflammatory monocyte mobilization and enhance tissue injury [102]. Depletion of B cells by rituximab (CD20 specific antibody) had beneficial effects after MI [102]. The precise role of HMGB1 in the context of B cells regulation in the inflammatory reparative response has not been fully delineated. HMGB1 has been demonstrated to affect B cells activation in response to endogenous TLR9 ligands [103] and through TLR2 and CD36 in inflammatory bowel disease [104]. Moreover, a recent study has demonstrated that the HMGB1–CXCL12 complex influences B-cell trafficking in Peyer’s patches (PPs) and IgA production in the intestine [105], and, even if this role was observed in homeostatic condition, it is possible that HMGB1 may exert similar effects during acute or chronic inflammation. All these data may suggest a possible role of HMGB1 in the regulation of B cells during the inflammatory reparative response that has not been investigated yet.

T lymphocytes are a key component of the adaptive immune system and can be divided into helper T cells (CD4^+^), cytotoxic T cells (CD8^+^), and regulatory T cells (Treg). T cells are activated within few days after MI in lymph nodes draining the myocardium [106]. Patients with MI had lower CD4^+^, but higher CD8^+^ T lymphocytes [101], leading to a depressed CD4/CD8 ratio that, if prolonged, is considered a poor prognostic factor [101,107]. Conversely, in vivo studies reached opposite results: CD4^+^ T cell deficient animals had a smaller infarct size after MI compared to wild-type animals [108]. Accordingly, adoptive transfer of CD4^+^ T cells in Recombination activating gene 1 knockout (RAG1-KO) mice, which are deficient in lymphocytes, blunted the protective effect due to the depletion of CD4^+^ T cells [109]. In a murine model of isoproterenol-induced myocardial ischemia, it has been recently showed that exogenous HMGB1 treatment aggravated myocardial injury and increased both the CD4/CD8 ratio and the expression level of interleukin-17 (IL-17) compared to untreated infarcted mice. These effects were mediated by TLR4 since myocardial ischemic injury in TLR4 knockout mice was alleviated and the CD4/CD8 ratio and IL-17 expression level were both reduced [110].

Regulatory T cells (Treg) are a CD4^+^/CD25^+^ subset of T lymphocytes, centrally involved in maintaining self-tolerance and suppress aberrant or excessive immune response [111]. Animal studies indicated that depletion of Treg after MI leads to a loss of regulation of the immune system, which, in turn, triggers and amplifies an exaggerated inflammatory response, thus worsening LV remodeling; as confirmation, Treg injection was found to decrease infarct size and improve cardiac function after MI [92] and activation of Treg by superagonistic anti-CD28 monoclonal antibody administered 2 days after MI improved healing and survival [112]. There is evidence showing that Treg may play a role in suppression of the post-infarction inflammatory response [113]: following MI, Treg infiltrate the healing myocardium, where they induce the differentiation of M2 macrophages and secrete anti-inflammatory factors, like IL-10 and TGF-β, to inhibit the inflammatory response of M1 macrophages and lymphocytes, thereby ameliorating inflammation-mediated cardiac damage [112,114]. Moreover, Treg changed the phenotype and function of cardiac fibroblasts and decreased excessive ECM degradation, thus alleviating myocardial fibrosis and cardiac remodeling after MI [112,114,115]. For all the considerations above, Treg cells could be a promising key target for the immunomodulation of MI.

HMGB1 has been demonstrated to regulate the proliferation, functions, and homeostasis of regulatory T cells. In the context of tumor, HMGB1 has a chemoattractant role for Treg, promotes their survival and enhances their immune inhibitory functions [116]. However, it has also been reported that, in vitro, HMGB1 stimulation induced a downregulation of Treg phenotype [117]. To date, the role of HMGB1 in regulating Treg in the context of the inflammatory reparative response following MI has not been evaluated. However, based on the data obtained in other pathological conditions, it is conceivable that this regulation could have an important role also in this context.

## 4. Other Mechanisms of HMGB1-Mediated Cardiac Repair and Regeneration after MI

Several studies have demonstrated that HMGB1 mediates cardiac tissue regeneration following injury by targeting endogenous stem cells [118,119] through mechanisms not yet completely elucidated and still controversial. Interestingly, recent reports demonstrated that stem cell therapy could modulate the local inflammatory response in the damaged heart. For instance, cardiosphere-derived cells are able to modulate the inflammatory state of macrophages from pro-inflammatory to anti-inflammatory within the myocardium leading to a long-term improvement in cardiac function [120]. More recently, Kang and colleagues found that cardiac mesenchymal cells (CMCs) exert immunomodulatory action on neutrophils and macrophages [121]. In particular, they hypothesized a contribution of myocardial neutrophil infiltration to CMC-mediated cardiac repair possibly by a shift of neutrophils towards an anti-inflammatory and reparative phenotype. In the setting of I/R, macrophage depletion using clodronate liposomes abolished the protective effects of fractionated bone marrow mononuclear cells (MNCs) treatment after I/R while intramyocardial injection of MNCs induced local CCR2^+^ and CXCR1^+^ macrophage accumulation and provided functional improvement [122]. Therefore, it might be possible that HMGB1-mediated activation of resident cardiac stem cells provides beneficial effects via an immunomodulatory mechanism, i.e., increasing the recruitment of immune cells.

HMGB1 also contributes to cardiac regeneration affecting cardiac fibroblasts. HMGB1 has been suggested to promote cardiac regeneration via a paracrine mechanism mediated by cardiac fibroblasts [123]. These cells, following HMGB1 treatment in vitro, increased the production of growth factors, cytokines, and chemokines that induced resident cardiac c-kit^+^ cell migration, proliferation, and differentiation toward the endothelial phenotype [123]. In vivo, using a murine model of heart failure, intramyocardial HMGB1 injection attenuated LV remodeling most likely through the inhibition of TGF β/Smad signaling pathway known to play an important role in the pathogenesis of cardiac remodeling and fibrosis [124]. Nevertheless, a recent study demonstrated that, in a mouse model of cardiac fibrosis induced by subcutaneous injection of isoproterenol, HMGB1, interacting with its receptor TLR2, stimulated fibrosis by suppressing fibroblast autophagy [125].

Interestingly, it has been demonstrated that immune cells and cardiac fibroblasts influence each other in the infarcted myocardium. A recent study suggested that in this context fibroblast-derived GM-CSF might play an important function in chemotactic attraction of neutrophils and monocytes [126]. On the other side, several subpopulations of lymphocytes [115] and mast cells [127] play an important role in the activation of cardiac fibroblasts. For instance, Treg are known to modulate the phenotype of cardiac fibroblasts and their function: in a mouse model of I/R injury, Treg exerted protective effects that resulted in attenuated adverse cardiac remodeling both by suppressing pro-inflammatory mediator expression and reducing the matrix degrading activity of fibroblasts [115]. It is noteworthy that in the tumor microenvironment, HMGB1 has been demonstrated to induce migration and prolong survival of Treg. According to all these findings, it would be interesting to verify whether, in vivo, the effects of HMGB1 on cardiac fibroblasts are mediated by immune cells.

Importantly, HMGB1 promotes angiogenesis. Since inflammation and angiogenesis are closely inter-related processes, also several components of the immune system are key players in the process of neovascularization.

Neutrophils play an important role in angiogenesis through different mechanisms. One is the regulation of vascular repair through AMP-activated protein kinase α2 (AMPKα2), which promotes the generation of pro-angiogenic factors (such as VEGFA and VEGFB) [128]. Furthermore, it has been demonstrated that VEGFA, released under ischemic conditions, promotes the recruitment of a specific subset of circulating VEGFR1high CXCR4high neutrophils with high MMP9 expression levels, to facilitate rapid angiogenesis at hypoxic areas [129,130]. However, some evidence suggests that neutrophils could secrete factors that restrain the angiogenic process. For instance, neutrophilic elastase induces EC apoptosis [131]. Moreover, neutrophils are also a major source of ROS, which can induce EC apoptosis, as well [132]. Along this line, neutrophils are major mediators of microvascular dysfunction after MI and, notably, promote ischemia/reperfusion injury and the no-reflow phenomenon [133,134].

Monocytes/macrophages are the best described regulators of angiogenesis. Circulating monocytes can promote angiogenesis through the secretion of different growth factors, such as FGF-2 and VEGF, or proteases, such as MMP-9 [74]. It has been demonstrated that the non-classical CD14^+^CD16^+^ monocytes produce higher levels of VEGF in the mouse ischemic myocardium than the classical phenotype, suggesting a stronger pro-angiogenic activity [75]. Moreover, cell sorting analyses showed that CD14^++^CD16^+^ monocytes expressed the highest levels of CXCR4 [135], Tie2, and VEGFR2 [136] on their membrane, when compared to other subtypes, highlighting the importance of intermediate populations in post-MI angiogenesis. Monocytes recruited into ischemic tissues can also function as angioblasts, acquiring endothelial-like properties after angiogenic stimulus [137]. These cells, known as endothelial progenitor cells, can adhere to the endothelium at sites of ischemia and participate in new vessel formation. Early infiltrating inflammatory macrophages initiate the angiogenic process, co-localizing with EC tips. These cells are replaced by reparative macrophages which promote neovascularization through the release of pro-angiogenic factors such as insulin-like growth factor-1 (IGF-1) and CCL2 [138]. M2 macrophages show high expression levels of matrix metalloproteinase 9 (MMP9) and are thought to promote angiogenic functions through the release of proangiogenic factors and to induce tissue repair and vascular remodeling [74]. Indeed, intravenous injection of M2 macrophages into mice immediately after coronary artery ligation determines an improvement in cardiac neovascularization [139]. M1 macrophages may have a positive role in angiogenesis as well, although to a different extent: it has been shown that both M1 and M2 macrophages produce MMP-9, but M2 macrophages also display a reduced expression of tissue inhibitor of metalloproteinase 1 (TIMP-1), therefore showing an angiogenesis-inducing capacity higher than that one of M1 macrophages [140]. Nevertheless, monocytes isolated from SHIP^−/−^ mice (a gene that induces M2 polarization preferentially) after ischemia are not effective in promoting post-ischemic angiogenesis. Furthermore, paracrine signaling may participate in macrophage pro-angiogenic functions. Activated ECs secrete angiopoietin-2 (Ang2), which binds Tie2-expressing monocytes/macrophages, enhancing their angiogenic potential [141]. All these findings point to monocytes/macrophages as key regulators of angiogenesis.

HMGB1 induces angiogenic responses directly by mediating angiogenic cytokine release and indirectly by inducing other proangiogenic cells, including macrophages [142]. Specifically, HMGB1 could stimulate the recruitment and stimulation of macrophages that, as described before, promote angiogenesis through the secretion of different angiogenic factors, such as FGF-2, TGF β1, and VEGF, or could also function as angioblasts, acquiring endothelial-like properties after angiogenic stimulus [143]. In the heart, several studies have demonstrated that HMGB1 induces angiogenesis after injury by upregulating the expression of VEGF [144,145]. Whether VEGF release is induced directly or indirectly by HMGB1 has not been investigated.

In a rat model of permanent ligation, systemic administration of HMGB1 for 4 consecutive days led to the formation of new vessels and reduced fibrosis by the recruitment of PDGFRα^+^ bone marrow-derived mesenchymal stem cells (BM-MSC) from the bone marrow via CXCR4/SDF1 signaling. All these effects inhibited adverse LV remodeling leading to an improvement in cardiac function. The authors hypothesized that PDGFRα^+^ BM-cells might have secreted various growth factors such as VEGF in the damaged myocardium and some might have differentiated into vessel cells such as vascular endothelial cells or pericytes, in the peri-infarcted area [146]. The authors revealed that SDF1 expression was significantly increased in MI rats, particularly in the peri-infarction area. It cannot be excluded that this increase might have induced the recruitment not only of BM-MSCs but also of macrophages via CXCL12/SDF1 signaling complex.

## 5. Extracellular Functions of Redox Forms of HMGB1 following Injury

All HMGB1 functions are mainly influenced by its posttranslational modification. Specifically, in the last years, it has become evident the importance of HMGB1 redox state that depends on the microenvironment. In order to better understand the role of the various redox forms of HMGB1 in different contexts, several HMGB1 recombinant proteins have been produced (Figure 1B). By using these recombinant proteins, and it has been demonstrated that: (1). in its reduced state (fr-HMGB1), HMGB1 is involved in the recruitment of inflammatory cells (e.g., monocytes and leukocytes) and in tissue regeneration; (2). in its disulfide form (ds-HMGB1), it exerts cytokine-inducing activity (reviewed in [41]); (3). while when fully oxidized (ox-HMGB1), it promotes immune tolerance [147]. These diverse biological activities are determined by the binding of HMGB1 to different proteins, in particular receptors, that in turn is influenced by HMGB1′s oxidation status. In particular, fr-HMGB1 can interact with RAGE and CXCR4 [44], while ds-HMGB1 activates TLR2/4 [148].

In the context of myocardial infarction, fr-HMGB1, once released in the oxidizing environment generated after MI by ROS production, becomes disulphide HMGB1 first and ox-HMGB1 later [149]. Fr-HMGB1 induces cardiac fibroblast and stem cell migration through Src activation while ds-HMGB1 stimulates pro-inflammatory cytokine secretion in macrophages via TLR4; ox-HMGB1, instead, has no apparent activity [41]. Interestingly, the interplay between fr-HMGB1 and ds-HMGB1 is a reversible process while ox-HMGB1 is irreversibly transformed and this progressive oxidation of the protein is fundamental to correctly coordinate the functions of all the cellular effectors involved in the inflammatory and reparative response following myocardial infarction. The mutant form of HMGB1, i.e., 3S, that mimics fr-HMGB1 functions in vitro and cannot be converted into other redox forms, has been used to study HMGB1 functions following MI, a pathology with an oxidizing context (Figure 1C) [149].

Using an in vivo model of MI, 3S intramyocardial injection induced cardiac fibroblast migration more efficiently than fr-HMGB1 and at a lower concentration, indeed [149]. This effect was CXCR4-dependent but did not require CXCL12. 3S determined conformational changes in CXCR4 that were different from those induced by CXCL12 and this direct CXCL12-independent CXCR4 activation could explain the higher effectiveness of 3S in inducing cardiac fibroblast migration and Src activation compared to fr-HMGB1. Nevertheless, by inducing sustained fibroblast migration, 3S treatment elicited detrimental effects compared to fr-HMGB1, resulting in adverse LV remodeling and worsening of cardiac dysfunction. Interestingly, cardiac remodeling was induced not only by excessive collagen deposition due to an increase in myofibroblast number, but also by a lack of compensatory hypertrophy and neo-angiogenesis. It should be noted that several previous studies reported that while CXCL12/CXCR4 axis exerts protective effects after MI, CXCR4 signaling may have pleiotropic effects in the ischemic heart being protective by promoting angiogenesis [150], attenuating cardiomyocyte apoptosis [151], and enhancing the regenerative capacity of mobilized progenitor cells [152] or detrimental (when overexpressed in the infarcted heart by adenovirus-mediated gene therapy or using heterozygous Cxcr4^+/−^ mice) by enhancing recruitment of inflammatory cells and activating pro-apoptotic pathways [153,154].

Aside from the mechanism underlying 3S-mediated adverse cardiac remodeling after MI, all these findings confirmed the hypothesis that the progressive oxidation of the protein is fundamental to correctly coordinate cardiac fibroblast functions and, therefore, tissue healing after injury. Accordingly, one of the very first studies investigating the effect of exogenous HMGB1 in a murine model of MI demonstrated that intramyocardial administration of fr-HMGB1 was able to mediate repair and regeneration leading to an improvement in cardiac function [155]. These effects were mediated by enhanced cardiomyocyte survival and activation of resident stem cells. Unfortunately, in the study by Di Maggio and colleagues, the authors did not examine resident cardiac stem cells in the infarcted treated heart. Surprisingly, four weeks after MI, they did not have significant functional improvements neither by echocardiography nor by hemodynamic analysis (only after 1 wk but not after 2 and 4 wk) following injection of fr-HMGB1 compared to controls, even though they adopted an identical protocol and the same time points for functional measurements present in the study by Limana et al. [155]. These findings are also in contrast with the enhancement in neo-angiogenesis that the authors detected at the same time point. By morphometric analysis, they measured an increased wall thickness of the infarcted segment in fr-HMGB1 treated mice compared to vehicle- and 3S-treated mice claiming that this increase was due to the presence of viable cardiomyocytes surrounding the infarcted area but without specifying whether these cardiomyocytes were newly formed ones from resident stem cells or surviving cardiomyocytes, instead. Finally, they did detect adaptive hypertrophy only in vehicle-treated hearts but not in 3S-treated hearts and it is quite strange that an infarcted heart with deterioration in function does not present any compensatory mechanism.

It is of interest that the same mutant form, i.e., 3S, elicited completely different results in muscle and liver injury [156] supporting regeneration more efficiently than fr-HMGB1 and without the need, as for fr-HMGB1, to form a complex with CXCL12.

In the in vivo model of muscle injury, indeed, the authors demonstrated that both fr-HMGB1 and 3S supported muscle repair and regeneration without inducing inflammation. Accordingly, they modulated macrophage polarization toward a tissue-healing phenotype as showed by a significant increase in the number of CD163^+^ tissue-healing macrophages and an enhancement in the expression of IGF-1, a growth factor known to promote macrophage polarization toward the tissue-healing phenotype [157,158,159] in treated muscles compared to controls. It is interesting that a very recent report reached completely different results by comparing the effects of ds-HMGB1 and fr-HMGB1 on the polarization of murine bone-marrow-derived macrophages (BMDMs) [160]. Specifically, they found that fr-HMGB1 did not polarize BMDMs while ds-HMGB1 was able to induce an M1-like phenotype, i.e., a pro-inflammatory phenotype, that, anyway, is different from the classic M1 induced by LPS/IFN-γ. Further, both redox isoforms induced BMDM migration but ds-HMGB1 by binding to TLR4 while fr-HMGB1 via other receptors.

In conclusion, in both studies by Di Maggio et al. and by Tirone et al., 3S directly interacts with CXCR4 and is more effective than fr-HMGB1 but in the context of MI induced by coronary artery ligation (HMGB1 treatment 4 h after MI) it exerts detrimental effects by affecting fibroblasts while in the in vivo model of muscle injury induced by cardiotoxin injection (HMGB1 treatment simultaneously with cardiotoxin injection) it triggered tissue regeneration by promoting satellite cell migration/proliferation and hepatocyte proliferation without inducing inflammation as demonstrated by macrophage polarization toward a tissue-healing phenotype. It would be interesting to investigate whether (1) the detrimental effects in the infarcted heart are mediated directly by fibroblasts or by immune cells activated by fibroblasts and (2) if the beneficial effects in the injured skeletal muscle exerted by satellite cells are direct or due to anti-inflammatory macrophages

## 6. Conclusions

The inflammatory response following MI is essential for the reparative process to occur. However, only a flawless orchestrated response, involving several cell components of the immune system, could secure an optimal healing of the myocardium. HMGB1, acting as a DAMP, plays a fundamental role in this context. Specifically, extracellular HMGB1 firstly induces monocyte recruitment to the site of injury and induces them to secrete inflammatory cytokines. Nevertheless, HMGB1 also supports cardiac tissue repair by coordinating the switch of macrophages to a tissue-healing phenotype and suppressing DC cytokines secretion and accumulation. Aside from its effects on cardiac healing by direct modulation of immune cells, HMGB1 has been demonstrated to contribute to cardiac repair and regeneration by activating stem cells, endothelial cells, and endothelial progenitor cells and, also, by affecting fibroblasts.

In recent years, it has become evident that the activity of HMGB1 is strongly influenced by its post-translational modifications. In particular, in the context of MI, the oxidizing environment generated by ROS production could have an important effect on the redox status of HMGB1 and, therefore, on the regulation of its functions. Further studies will be needed to have a complete understanding of the role of HMGB1 in orchestrating the immune compartment in the complex process of the inflammatory reparative response following MI.
